# Imported Human Fascioliasis, United Kingdom

**DOI:** 10.3201/eid1511.090511

**Published:** 2009-11

**Authors:** Meera A. Chand, Joanna S. Herman, David G. Partridge, Kirsten Hewitt, Peter L. Chiodini

**Affiliations:** Hospital for Tropical Diseases, London, UK (M.A. Chand, J.S. Herman, P.L. Chiodini); Royal Hallamshire Hospital, Sheffield, UK (D.G. Partridge); Health Protection Agency Centre for Infections, London (K. Hewitt); London School of Hygiene and Tropical Medicine, London (P.L. Chiodini)

**Keywords:** Fasciola spp., fascioliasis, zoonosis, khat, parasites, eosinophilia, United Kingdom, letter

**To the Editor:** We initiated enhanced surveillance for human fascioliasis after a reported increase in livestock cases in the United Kingdom. From January 1, 2008, through January 31, 2009, 11 human cases were confirmed by the reference laboratory for England and Wales, compared with 6 cases during the preceding 10 years. The Scottish reference laboratory detected no human cases during the study period.

Fascioliasis was defined as a positive *Fasciola* immunofluorescent antibody test with a screening titer of 1:32 and either compatible clinical or radiologic features consistent with the disease. We obtained clinical and radiologic information from the referring physician. Clinical features of both acute and chronic infection include fever, upper abdominal pain, malaise, eosinophilia, and impaired liver function; therefore, distinguishing between the 2 phases can be difficult. Fifty percent of chronic infection is subclinical (*1**,**2*). Compatible radiologic features are capsular enhancement with contrast, hypodense nodular areas, and low-density serpiginous lesions (*2*). Our analysis comprised 11 cases (Table). Two patients were white British, both of whom had recently traveled to sub-Saharan Africa. Cases from the preceding 10 years diagnosed in our laboratory were all in persons with histories of travel to fascioliasis-endemic areas. Therefore, these cases do not provide firm evidence of indigenous zoonotic transmission within England and Wales.

**Table:** Characteristics of human fascioliasis case-patients during enhanced surveillance, United Kingdom, January 1, 2008–January 31, 2009*

Case no.	Age, y/sex	Country of origin	Years since migration	Other travel	Risk factor	Clinical features	Eosinophil count, x10^9^/L)	Abnormal liver function	Hepatic imaging	IFAT†
1	45/F	Yemen	7	Yemen regularly	*Khat* use	Abdominal pain	8.4	Yes	Mixed-density liver lesion (CT)	1:128
2	44/M	Somalia	16	Ethiopia 2007	*Khat* use	Fever, abdominal pain	3.4	Yes	Serpiginous lesion (MRI)	1:64
3	34/F	Ethiopia	3	S. Africa regularly	*Khat* use	Fever, abdominal pain	11.4	No	Heterogeneous lesion (USS)	1:128
4	44/F	Somalia	7	Somalia 2004, Netherlands	*Khat* use	Abdominal pain	8.3	No	Heterogeneous lesion (USS)	1:128
5	54/F	Somalia	21 (to Netherlands), 4 (to UK)	None	*Khat* use	Anorexia	8.4	No	Low-density lesion (CT)	1:32
6	43/M	Somalia	28 (to India), 21 (to UK)	None	*Khat* use	Fever	1.0	Yes	Heterogeneous lesion (USS)	1:128
7	28/F	UK	–	Uganda 2007–2008	–	Abdominal pain, hepatomegaly	1.84	Yes	Hepatomegaly with large mixed cystic and solid lesion (USS)	1:512
8	67/M	UK	–	Kenya 2008, prior world travel	–	Malaise, abdominal pain	0.04	Yes	Multiple gallstones (MRCP)	1:256
9	38/M	Ethiopia	10	Ethiopia 2006	–	Abdominal pain, fever	18.7	Yes	Normal (USS, MRCP)	1:128
10	28/M	Ethiopia	Unknown	Unknown	–	Fever, gram- negative sepsis; new HIV diagnosis	<0.04	Yes	Lesion in hepatic vein	1:64
11	47/F	Somalia	16 (to Yemen), 6 (to UK)	Unknown	*Khat* use	Abdominal pain, fever	16.8	Yes	Low-density lesion (CT)	1:256

*IFAT, immunofluorescent antibody test; CT, computed tomography; MRI, magnetic resonance imaging; USS, ultrasound scan, MRCP, magnetic resonance cholangiopancreatography. †Titer of IFAT (screening titer 32).

Nine patients originated from Somalia, Ethiopia, or Yemen. Few cases have previously been reported from this area (*3*), although Ethiopian migrants have been shown to have an egg positivity of 0.4% on routine screening (*4*). Patients 5 and 6 had not returned to Africa for >20 years, suggesting that they acquired their infection in Europe. Therefore, a risk factor may exist that is specific to this ethnic group within the United Kingdom.

Six cases were diagnosed at 1 hospital. All 6 patients reported current or past use of locally bought *khat*, a leaf chewed for its stimulant properties. It is imported fresh to the United Kingdom from Africa and is an ideal environment for the survival of *Fasciola* cercariae. It is used most commonly by migrants from the Horn of Africa and Yemen and has been reported in association with acute fascioliasis in the United Kingdom (*5*). Use of imported khat may explain the apparently higher incidence of fascioliasis in this ethnic group residing in the United Kingdom.

Despite the described parallel rise in human and veterinary fascioliasis, none of these cases provide clear evidence that recent human cases resulted from zoonotic transmission within the United Kingdom. Most cases occurred in migrants from the Horn of Africa and Yemen, some of whom may have acquired *Fasciola* spp. in their country of origin; other cases appear likely to have been acquired in the United Kingdom, possibly due to use of imported *khat*. Physicians need a heightened awareness of fascioliasis when investigating impaired liver function or abnormal abdominal imaging in migrants or travelers from high-risk areas.

## References

[1] Marcos LA, Terashima A, Gotuzzo E. Update on hepatobiliary flukes: fascioliasis, opisthorchiasis and clonorchiasis. Curr Opin Infect Dis. 2008;21:523–30. 10.1097/QCO.0b013e32830f9818 18725803

[2] Marcos LA, Tagle M, Terashima A, Bussalleu A, Ramirez C, Carrasco C, Natural history, clinicoradiologic correlates and response to triclabendazole in acute massive fascioliasis. Am J Trop Med Hyg. 2008;78:222–7. 18256419

[3] Control of foodborne trematode infections. Report of a WHO study group. World Health Organ Tech Rep Ser. 1995;849:1–157. 7740791

[4] Nahmias J, Greenberg Z, Djerrasi L, Giladi L. Mass treatment of intestinal parasites among Ethiopian immigrants. Isr J Med Sci. 1991;27:278–83. 2050509

[5] Doherty JF, Price N, Moody AH, Wright SG, Glynn MJ. Fascioliasis due to imported khat. Lancet. 1995;345:462. 10.1016/S0140-6736(95)90450-6 7853987

